# Effectiveness of self-management programmes for athletes with patellofemoral pain syndrome: A systematic review

**DOI:** 10.17159/2078-516X/2025/v37i1a18648

**Published:** 2025-02-15

**Authors:** A Masoudi, N Chemane, N Magida, U Useh, B Bello

**Affiliations:** 1 Department of Physiotherapy, School of Health Sciences, University of KwaZulu-Natal, Durban, South Africa; 2 Senior Physical Therapist King Fahad Hospital, Rehabilitation Department, Al Madinah, Saudi Arabia; 3 Department of Physiotherapy, Faculty of Healthcare Sciences, University of Pretoria, Pretoria, South Africa; 4 Lifestyle Disease Research Entity, Faculty of Health Sciences, Northwest University, Mafikeng Campus, South Africa; 5 Physiotherapy Department, Faculty of Allied Health Sciences, Bayero University, Kano, Nigeria

**Keywords:** sports performance, functional ability, home programme, musculoskeletal disorders

## Abstract

**Background:**

Patellofemoral pain syndrome (PFPS) is a common condition that can severely hinder an athlete’s ability to perform and compete at their full potential and overall well-being. The emergence of self-management programmes presents a potential treatment avenue for athletes with PFPS. Self-management programmes aim to empower patients through education, symptom monitoring, tailored exercises, and gradual activity progression while respecting pain thresholds. Despite their potential benefits, limited data evaluates the effectiveness of self-management programmes specifically tailored for athletes with PFPS.

**Objectives:**

To assess the effectiveness of self-management programmes in improving pain and functional ability among athletes with PFPS.

**Methods:**

A systematic search was conducted across PubMed/MEDLINE, Cochrane Library, CINAHL, and PsycINFO databases. Randomised and non-randomised controlled trials comparing self-management interventions to other treatments or control groups for improving pain and functional ability among athletes with patellofemoral pain syndrome were included. Studies including athletes with PFPS (defined as pain persisting for ≥12 weeks) were eligible. Four reviewers independently extracted data and assessed quality using the Physiotherapy Evidence Database (PEDro) scale.

**Results:**

Three trials involving 139 participants met the eligibility criteria. Identified self-management programmes comprised exercises, mindfulness training, and educational programmes tailored to athletes. Two of the eligible studies identified found that exercise consisting of the combination of mindfulness and/or dry needling reported an enhanced recovery rate in the studied population with regards to PFPS. The other study reported no added benefit whether exercise or education was used as a modality for the self-management of PFPS.

**Conclusion:**

While some studies showed promising results for self-management programmes involving exercises and mindfulness in managing patellofemoral pain syndrome among athletes, the overall evidence could be more extensive and consistent. High-quality controlled trials with consistent methodology and athletic populations are needed to identify the most effective self-management approaches for patellofemoral pain syndrome in athletes.

Athletes often face physical discomfort and injury challenges during training and competitions.^1^ Among these issues, patellofemoral pain syndrome (PFPS) is a prevalent condition that frequently leads to pain experienced in the anterior region of the knee, especially among athletes involved in constant running, cycling, jumping, cutting, and pivoting.^2^ The prevalence of PFPS ranged from 25% to 40% among all knee injuries.^3^ Patellofemoral pain syndrome worsens during activities such as squatting, climbing stairs, running, jumping, cycling, or remaining seated for extended periods with the knees bent.^4^ The aetiology of PFPS is complex, multifactorial, and not yet fully understood. Nonetheless, research suggests it likely involves local factors around the knee joint and proximal factors related to the hip and pelvis.^5^ Certain intrinsic risk factors may predispose an individual to develop PFPS.^6^ These include a delayed activation timing of the vastus medialis obliquus muscle relative to the other quadriceps muscles and weakness in the hip abductor and external rotator muscle groups.^6^ In addition, there is an imbalance in strength among the quadriceps muscles, hamstrings, iliotibial band, or calf muscles, excessive inward rolling of the feet, and a knock-kneed alignment.^6^ Extrinsic risk factors like improper training regimens, inappropriate footwear, and running on uneven surfaces may also contribute to PFPS.^7^ Despite similar clinical presentations, recent evidence suggests that PFPS may represent several distinct subtypes requiring targeted interventions. Selhorst, Fernandez-Fenandez, Schmitt and Hoehn^8^ proposed four subtypes of PFPS: mal-tracking, instability, overload, and hypoalgesia. The mal-tracking subtype exhibits excessive lateral patellar tilt and shift during knee flexion. The instability subtype displays recurrent patellar dislocations or subluxations, and the overload subtype shows no obvious malalignment but increased patellofemoral joint stress from overuse.^8^ The hypoalgesia subtype has delayed onset muscle soreness but reduced patellofemoral joint tenderness on palpation.^8^ Identifying clinical findings consistent with a specific PFPS subtype allows customised treatment programmes.

Identifying susceptible individuals and addressing modifiable risk factors is crucial in preventing PFPS among athletic populations. In athletes diagnosed with PFPS, tailored rehabilitation programmes focused on correcting lower extremity muscle imbalances, flexibility deficits, movement impairments, and gradual return to sport are needed for optimal recovery and reduced recurrence.^9^ First-line treatment generally includes patient education, activity modification, quadriceps strengthening, hip and core musculature strengthening, flexibility exercises, patellar taping or bracing, and gradual return to activity.^10^ Given the multifactorial nature of PFPS, a variety of adjunctive therapies are also being explored to complement traditional rehabilitation. Patellar taping techniques, like the McConnell tape, have been utilised to help correct faulty patellar tracking and reduce pain by improving patellofemoral biomechanics.^11^ Instrument-assisted soft tissue mobilisation, dry needling, massage, and other manual therapies may help address restricted tissue mobility around the knee.^12^ Orthotics and gait retraining can help correct overpronation and abnormal movement patterns.^13^ Deficits in hip strength, especially the abductors and external rotators, have been associated with PFPS.^14^ Programmes incorporating resistance training for the hip musculature demonstrate reduced pain and improved function in PFPS patients with hip weakness.^15^ Stretching and muscle energy techniques for tight muscles like the iliotibial band, quadriceps, and gastrocnemius also have the potential to relieve symptoms.^16^ Correcting muscle imbalances with exercises targeting the vastus medialis obliquus may help address patellar maltracking tendencies.^17^ Addressing identified movement impairments and integrating individualised multi-modal interventions tailored to deficits seems essential to effective PFPS rehabilitation.

Recently, self-management programmes (SMP) have emerged as a promising alternative model of care for athletes with PFPS.^18^ These programmes aim to empower patients to actively manage their condition via education, symptom monitoring, individualised exercise, and progressing activity levels within pain limits.^18^ A systematic review recently investigated the impact of taping on PFPS, and the findings indicate low-quality evidence suggesting that taping could offer short-term pain relief for PFPS patients.^19^ However, the authors underscored insufficient high-quality evidence supporting taping’s effectiveness in improving function, strength, range of motion, and biomechanics among PFPS patients.^19^ The review accentuates the conflicting and diverse findings in current research regarding the use of taping for PFPS. Another study found very low-quality but consistent evidence that exercise may clinically improve pain and function for PFPS patients.^20^ The author concluded that the studies’ heterogeneity and poor methodological quality precluded determining the optimal type of exercise for PFPS management.^20^ All these previous systematic reviews on PFPS did not target athletes or interventions specifically used as self-management programmes.^[19,20]^ Thus, the generalisability of the findings to all PFPS patients, including athletes, would be misleading and inappropriate for the population. Hence, there is a need for a comprehensive study synthesising the current evidence on the effectiveness of SMPs in managing PFPS among athletes. The findings from this systematic review will enable a thorough critical assessment of the current body of research literature examining the use of SMPs for treating PFPS among athletic populations. Based on the available evidence, our goal is to determine which self-management approaches are most effective for reducing pain, improving function, and enabling return to sport in this population. Systematic reviews play a vital role in analysing and summarising findings across studies to derive insights that optimise clinical decision-making and outcomes.^21^ This present review aims to provide an enhanced understanding of the potential of SMPs to revolutionise the management of athletes with PFPS.

## Methods

### Project registration

The systematic review adhered to the Preferred Reporting Items for Systematic Reviews and Meta-Analyses (PRISMA) checklist, and the protocol was registered with PROSPERO under the registration number CRD42017053923. The rationale for the criteria choices explains how adherence to PRISMA guidelines and registration with PROSPERO ensure methodological rigor and consistency, enhancing the scope and focus of the review.

### Eligibility criteria

Randomised clinical trials and non-randomised trials written in English with full text in peer-reviewed journals were included. Studies including athletes with PFPS (defined as pain persisting for ≥12 weeks) were eligible. Studies evaluating SMPs as the primary intervention for PFPS were included. Self-management programmes included exercise therapy, muscle strengthening, psychotherapy, biofeedback, patient education, or a combination of these strategies. Studies with or without a comparator group were considered. Comparators included standard care, no intervention, placebo, or an alternative treatment for PFPS. Studies that assessed outcomes in terms of pain intensity, functional outcomes, and sports performance were included. Exclusion criteria included reviews, editorials, letters, commentaries, or protocol studies. Additionally, studies that considered participants who had undergone a surgical knee intervention were excluded.

### Search strategy

Systematic electronic search was done in CINAHL, PubMed, Cochrane Library, and PsycINFO electronic databases. The search included a combination of keywords and Medical Subject Headings (MeSH) terms related to PFPS, self-management programmes, functional ability, sports performance, and pain measurements. The Boolean operators “AND” and “OR” were utilised to refine the search strategy. A sample search strategy for PubMed/MEDLINE is outlined as follows; (“Anterior Knee Pain Syndrome” OR “Patellofemoral Syndrome” OR “Patellofemoral Pain” OR “Patellofemoral Pain Syndrome”) AND (“Self-management programme” OR “Exercise therapy” OR “Biofeedback” OR “Muscle strengthening” OR “Education” OR “Sports Performance”) AND (“Pain Intensity” OR “Pain Severity” OR “Pain Measurement” OR “Pain Assessment”). The search strategy was customised and adjusted for each database to thoroughly and extensively search the available literature.

### Selection process for identified studies

The study selection process adhered to a systematic and transparent approach, as outlined by Wright and colleagues.^22^ This involved multiple stages aimed at guaranteeing the inclusion of relevant and high-quality studies.

### Initial screening

Four independent reviewers (BB, AM, NC, and NM) initially screened titles and abstracts retrieved from the electronic databases based on the predefined inclusion and exclusion criteria. Studies not meeting the criteria were excluded during this stage. Two reviewers (BB and AM) independently obtained and evaluated the full texts of potentially relevant articles, judging them against the predetermined inclusion and exclusion criteria. In cases of disagreement or discrepancy between the two reviewers’ assessments, discussion took place among all authors until a consensus was reached to resolve the differing evaluations. We precisely handled our discrepancy as follows:

Two reviewers, BB and AM, disagreed on whether three articles met our eligibility criteria. BB excluded the studies, while AM included them. We held a Zoom meeting to resolve our differences but failed to reach a consensus. AM argued that the manuscripts included SMPs and involved athletes. BB contended that one study was a feasibility study with incomplete results, making it unsuitable for assessing the effectiveness of SMPs, the primary aim of our study. We invited two additional reviewers, NC and NM, to a subsequent Zoom meeting to resolve the issue. All four reviewers evaluated the original articles during this meeting and unanimously decided to exclude them. Two articles were protocols, and the third was an ongoing feasibility study without complete results to assess effectiveness.

### Data extraction process

Four reviewers (BB, AM, NC, and NM) independently conducted the data extraction process, ensuring a comprehensive and unbiased approach. Any disagreements or discrepancies during this process were addressed through open and constructive discussion among all the authors until a consensus was reached. The reviewers held regular meetings to discuss any uncertainties or inconsistencies in the data extraction and to resolve them collaboratively. The extracted data were cross-checked between reviewers to minimise errors and enhance reliability. Covidence software was used throughout the review process for screening and data extraction. The extracted data were saved and stored in Covidence, allowing easy access and retrieval during the synthesis and analysis stages. A version control system monitored updates or alterations during the data extraction phase. Attempts were also made to contact study authors to address any missing or ambiguous information. These procedures were implemented to bolster the reliability and validity of the systematic review’s findings, ensuring the highest standards of accuracy and thoroughness.

### Data synthesis

**Fig. 1:**
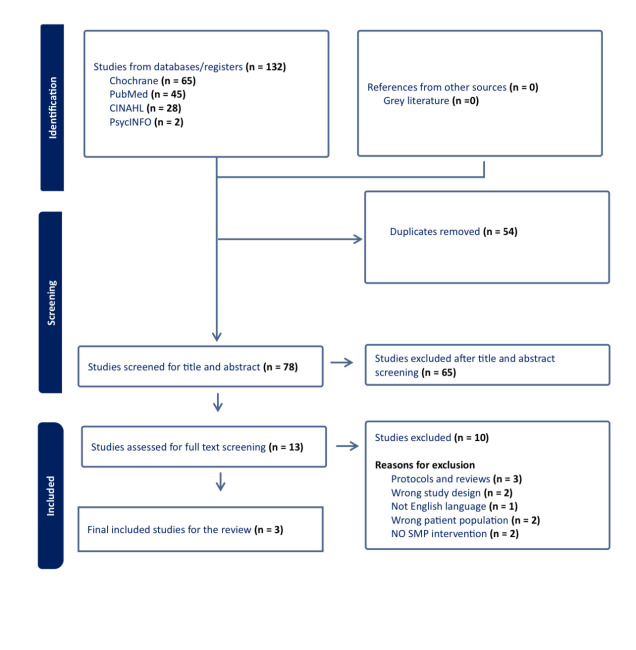
PRISMA Flow Chart Diagram. SMP, self-management programmes

The findings from the included studies were synthesised and summarised narratively, describing each study’s key characteristics and results. The extent of variation and inconsistency among the review’s included studies was evaluated by examining the variability in their study designs, participant populations, interventions implemented, and outcomes measured. The selection process, including the number of studies screened, reasons for exclusion at each stage, and the final list of included studies, were reported in a PRISMA flow diagram in Figure 1. A meta-analysis procedure was not carried out due to the lack of similarity of the outcome measures used across the included studies.

## Results

This systematic review aimed to evaluate and synthesise thoroughly the effectiveness of SMPs for improving outcomes in athletes with PFPS. Four electronic databases (PubMed, Cochrane, CINAHL, and PsycINFO) were systematically searched from inception to February 2024. The four databases obtained one hundred and thirty-two (132) records. After removing duplicates, 78 records were screened based on title and abstract, excluding 65 records that did not meet eligibility criteria. The remaining 13 full-text articles were assessed for eligibility, with ten (10) excluded for reasons including protocols only, interventions lacking self-management components, wrong population, and wrong study design. Ultimately, only three relevant trials with 139 participants met the full eligibility criteria and were included in the review (Figure. 1).

### Exercise intervention

All three included studies incorporated exercises in their SMPs. However, the specific exercise protocol varied in frequency, intensity, type, and duration. See Table 2 for details. Zarei and partners^23^ combined exercises with dry needling, while Bagheri and partners^24^ combined exercises and mindfulness training. In addition, Esculier and colleagues^25^ delivered a programme incorporating exercises, education, and gait retraining.

Esculier and colleagues^25^ implemented a standardised home exercise programme to improve strength, load-bearing capacity, and dynamic control of the lower limbs. The programme was personalised and progressed through two-week phases of increasing difficulty under physiotherapist guidance. Participants performed three to four exercises thrice weekly (maximum 20 minutes per session) and one daily lower limb control exercise^25^. However, the exact muscles targeted and specific exercise descriptions were not provided, limiting reproducibility. In contrast, the exercise protocol in Bagheri and partners^24^ was clearly described and reproducible and consisted of 13 exercises (six stretching, seven strength and balance) performed in three 60- to 90-minute weekly sessions for 18 weeks. Sessions included a 10-minute warm-up and cool-down with jogging and general dynamic exercises.^24^ Rest intervals were 30 seconds between sets and 90 seconds between exercises. The initial strength-training intensity was set at a 10-repetition maximum, producing pain ratings >3/10 on a visual analogue scale. Zarei and colleagues^23^ utilised a four-week progressive exercise protocol. Week one focused on hamstring and quadriceps stretching. Week two added isometric quadriceps strengthening in supine and side-lying positions. Week 3 progressed to include planking and side planking, while week four further advanced to mini-squats, mini-lunges, and step-downs. The exercises were well-described and reproducible.[Table t1-2078-516x-37-v37i1a18648]

In summary, while all three studies included exercise as part of their SMPs, there was significant heterogeneity in the specific exercise prescriptions utilised, as demonstrated in Table 2. This lack of uniformity highlights the need for further research to determine optimal exercise parameters within SMPs for managing patellofemoral pain syndrome in athletes.

### Educational intervention

In the study by Esculier and colleagues, runners received education on load management and were instructed to self-modify running training according to symptoms.^25^ Runners were asked to increase the frequency of their weekly training, decrease each session’s duration and speed, and avoid downhill and stair running. Run–walk intervals were allowed. Runners were instructed to maintain PFPS level at no more than 2/10 during running. Furthermore, pain had to return to pretraining levels within 60 minutes post-training without increased symptoms the following morning. Gradually, running distance was increased according to symptoms before adding speed and hills. The study concluded that the education-only group treatment outcome was not significantly different from combined education and exercise or gait training. Thus, the authors recommended that education on load management for athletes with PFPS be a core treatment component. Participants were also instructed on managing their training load and modifying their running according to their symptoms.^24^

### Mindfulness training

Incorporating mindfulness training into SMPs for athletes with PFPS presents a promising avenue for enhancing treatment outcomes. Bagheri and partners investigated the effects of mindfulness training as a component of SMPs for PFPS athletes.^24^ Participants in the mindfulness training group received instruction on various mindfulness meditation practices, including breathing exercises, body scan techniques, gentle yoga, and sitting and walking meditations. These practices aimed to cultivate greater awareness of thoughts, bodily sensations, and emotions, fostering an attitude of curiosity, openness, and acceptance.

The findings from Bagheri and partners revealed notable benefits associated with mindfulness training for PFPS athletes.^24^ Specifically, participants in the mindfulness training group reported reduced pain during activities such as running and stepping. They experienced fewer functional limitations in the knee than in the exercise-only group. These results highlight the potential of mindfulness-based interventions in alleviating symptoms and improving functional outcomes among individuals with PFPS. Integrating mindfulness training alongside conventional exercise regimens in self-management programmes holds promise for optimising the management of PFPS. By addressing not only the physical symptoms but also the psychological aspects of pain and discomfort, mindfulness training offers a holistic approach to treatment. Moreover, the findings underscore the importance of considering alternative modalities, such as mindfulness, in the comprehensive management of PFPS, ultimately enhancing the effectiveness of treatment approaches and promoting better overall well-being of athletes with this PFPS.

This systematic review generally found three experimental studies of moderate to high methodological quality investigating the effect of SMPs. Exercises, education, and mindfulness training were recommended for pain and functional ability in athletes with PFPS. Two studies found that exercises combined with either mindfulness or dry needling enhanced recovery versus comparison groups, while one study found no added benefit of exercises over education alone.

### Assessment of methodological quality

Each of the four reviewers independently evaluated the methodological quality and rigour of the studies included in the review, utilising the Physiotherapy Evidence Database PEDro Scale as the assessment tool (Table 3). Any disagreements in quality assessment were resolved through discussion and consensus.

The PEDro scale is an 11-item tool designed to assess the methodological quality of randomised controlled trials (RCTs) in physiotherapy and related fields. It includes items related to internal validity (e.g., random allocation, blinding) and statistical reporting (e.g., between-group comparisons). Each item is scored as either present (1) or absent (0), with a maximum possible score of 10 (the first item is not included in the total score). It is a widely recognised tool for assessing the methodological quality of systematic reviews, particularly in physiotherapy and related disciplines. The scale has been shown to provide a valid and reliable measure of the quality of trials included in systematic reviews, making it a valuable instrument for evaluating the robustness of evidence based practices.^26^ Its application in systematic reviews of physiotherapy trials has demonstrated sufficient reliability, as evidenced by its utilisation of high-quality reviews focusing on various interventions, such as injection treatments in tendinopathy.^27^ While initially developed for physiotherapy trials, the PEDro scale has expanded its utility to other areas of medicine, including nutrition and speech pathology, indicating its versatility and applicability across different disciplines.^28^ This adaptability underscores the scale’s effectiveness in assessing the methodological quality of diverse interventions and treatments, making it a valuable tool for researchers and practitioners seeking to evaluate the strength of evidence in systematic reviews. Moreover, the PEDro score has endorsed the QUOROM statement as providing sufficient reliability for systematic reviews of physiotherapy trials, further solidifying its credibility and acceptance within the research community.^29^ Its incorporation in systematic reviews of interventions for musculoskeletal diseases and other conditions highlights its importance in ensuring the methodological rigour and quality of evidence synthesis in healthcare research.^29^

In evaluating treatment effectiveness, the PEDro scale has been utilised to assess bias and research design quality, demonstrating its effectiveness in appraising the methodological robustness of studies included in systematic reviews.^30^ By considering factors such as blinding, randomisation, and sample size, the scale offers a comprehensive framework for evaluating the quality of research and informing evidence-based practice decisions. Its reliability, validity, and adaptability make it a preferred choicefor researchers conducting evidence synthesis and critical appraisal of interventions, ensuring the credibility and robustness of findings in healthcare and beyond.

## Discussion

In recent years, there has been growing interest in examining the efficacy of SMPs for reducing pain and enhancing functional abilities in athletes suffering from PFPS. This systematic review aimed to thoroughly evaluate and combine the current research findings on the effectiveness of SMPs as a treatment approach for PFPS syndrome among athletes. This systematic review synthesised the current evidence on the effects of SMP for athletes with PFPS. The review identified three interventional studies investigating different SMP components, including exercise, education, and mindfulness training, indicating the dearth of literature in the field of study.

The findings of this systematic review suggest that SMP incorporating a combination of exercise, education, and mindfulness training may be more effective for reducing pain and improving function in athletes with PFPS than individual components alone. One study found that combining exercise and trigger point therapy led to better outcomes than exercise alone.^23^ Additionally, mindfulness training combined with exercise was more beneficial than exercise alone.^24^ However, education on load management alone was equally as effective as education combined with exercise or gait retraining.^25^ The superior effects of multimodal SMP over individual components highlight the importance of a biopsychosocial approach for managing PFPS in athletes.^30^ Hence, targeting multiple factors involved in PFPS pathogenesis appears critical for optimal rehabilitation.

### Exercise intervention

Exercise intervention is crucial in the self-management of athletes with PFPS. Exercise aims to improve muscle strength and control around the patellofemoral joint. Exercise has emerged as a consistent component of effective SMP for PFPS across the studies.^31^ In their systematic review on the effectiveness of therapeutic physical exercise in treating PFPS, mentioned that numerous studies had demonstrated the effectiveness of exercise therapy in treating PFPS.^32^ Research has also shown that strengthening the hip abductor and lateral rotator musculature can significantly improve pain and function in female athletes with PFPS.^33^ Systematic reviews have further supported the benefits of exercise interventions, including quadriceps-strengthening exercises, in managing PFPS.^34^ Hence, improving muscular strength and control around the patellofemoral joint through targeted exercise may help address abnormal patellar tracking and overload, contributing to PFPS development.^35^ In particular, exercises focused on hip and thigh musculature seem beneficial for PFPS based on proposed mechanisms. Thus, reduced strength in the hip’s external rotator and abductor muscle groups can lead to excessive knee valgus and medial patellar displacement. At the same time, weak quadriceps increase patellofemoral joint stress,^36^ all leading to PFPS development. Strengthening hip external rotators and abductors muscle groups aims to optimise patellar alignment and reduce joint loads. The proximal muscle strengthening from exercise therapy may also help restore optimal movement patterns during functional activities like running. Altered kinematics and increased hip adduction during running are associated with a higher prevalence of PFPS in athletes.^37^ Improving strength and motor control through exercise can potentially normalise biomechanics during provocative activities. Exercise may also directly benefit pain perception in athletes with PFPS.^38^ Another study affirmed that resistance training helps decrease inflammation and induce endogenous pain inhibition by releasing betaendorphins and upregulation of serotonin.^39^ In addition, increased muscular fitness from exercise can also minimise pain by reducing relative joint loads during physical activity. Overall, the mechanical, neuromuscular, and analgesic effects of targeted muscle strengthening likely underpin the value of exercise therapy within SMPs for athletes with PFPS.^40^

### Exercise component

The findings of this review highlight the value of education as a component of SMPs for athletes with PFPS. Education focused on load management appears especially important for empowering athletes to effectively self-manage their symptoms. Educating athletes on modifying training in response to pain facilitates appropriate control of irritability and inflammatory processes associated with PFPS.^41^ Esculier and colleagues found that education on adjusting running speed, distance, rest intervals, and more, based on pain levels, was as effective as combining education with exercise or gait retraining. This result emphasises that athletes implementing load modifications through education alone can still experience significant improvements.^25^ Learning to reduce aggravating loads allows tissue healing while avoiding fear-avoidance behaviours that compound disuse weakness. Education could further enhance athletes’ understanding of PFPS aetiology and symptoms. Increased disease knowledge equips athletes to make informed decisions about their training regimens based on individual risk factors and presentation. Education may also promote adherence to other PFPS self-management behaviours such as exercise. Athletes informed on load management principles may be more motivated to perform rehabilitative exercises to return to sport safely.^42^ Overall, education is a low-cost intervention that gives athletes tools to take an active role in controlling PFPS symptoms. While education alone can be effective, combining it with exercise and psychosocial interventions may optimise outcomes. Regular educational sessions should be implemented at various time intervals to explain the nature of PFPS, the benefits of each exercise, and the importance of adherence. Also, providing written or digital materials outlining the SMP, including tips for maintaining adherence, would help ensure SMP effectiveness.

### Mindfulness training

Mindfulness training facilitates psychological coping and awareness of maladaptive movement patterns.^43^ The findings of Bagheri and colleagues suggest mindfulness training may provide added benefits when combined with exercise forathletes with PFPS.^24^ Incorporating mindfulness seems to target important psychosocial factors associated with chronic pain. Mindfulness techniques like meditation can help athletes alter maladaptive thought patterns that amplify pain perception. Catastrophising, fear-avoidance beliefs, and hypervigilance about pain are common cognitive distortions in chronic pain that mindfulness aims to address through non-judgmental awareness.^44^ Mindfulness also reduces pain-related anxiety and improves perceived coping ability. Athletes are then able to approach rehabilitation and training with greater psychological flexibility instead of avoiding activity due to kinesiophobia. Additionally, mindfulness facilitates body awareness critical for normalising dysfunctional movement patterns that underlie PFPS.^45^ Conscious control of muscle activation and mechanics is enhanced through mindfulness training. By modulating pain cognitions, emotions, and behaviors, mindfulness allows athletes to gain control over the sensory and affective dimensions of their PFPS.^46^ This facilitates active engagement in rehabilitation and long-term self-management. However, more research is required to determine the most effective format, dose, and content of mindfulness-based interventions for athletes with chronic pain conditions like PFPS. Integrating mindfulness practices such as meditation, breathing exercises, and body awareness techniques into athletes’ rehabilitation programmes, as well as psychological support to address pain-related anxiety and improve perceived coping ability would allow athletes to approach their rehabilitation with greater flexibility.

### External factors influencing SMP outcomes

While the components of SMPs are crucial, it is equally important to consider external factors that can significantly influence the outcomes of these programmes. These factors include training environments, athlete lifestyles, and adherence to self-management programmes. The training environment plays a vital role in the success of SMPs for athletes with PFPS. Optimal training environments should provide adequate equipment and space for performing prescribed exercises and facilitate proper technique execution.^48^ Additionally, supportive coaching staff and teammates can create a positive atmosphere that encourages adherence to SMPs.^48^ Conversely, high-pressure environments or those lacking proper facilities may hinder an athlete’s ability to implement self-management strategies, potentially compromising treatment outcomes consistently.^49^

An athlete’s lifestyle outside of their sport can significantly impact the effectiveness of SMPs. Sleep quality, nutrition, stress management, and overall work-life balance can influence an athlete’s ability to recover and respond to treatment.^50^ For instance, inadequate sleep has been associated with increased pain sensitivity and reduced pain tolerance, which could negatively affect PFPS management.^51^ Similarly, poor nutrition may impair tissue healing and recovery, potentially slowing progress in SMP outcomes.^52^ Addressing these lifestyle factors as part of a comprehensive SMP could enhance overall treatment efficacy.

Adherence to SMPs is a critical factor in determining their success. Self-management demands high self-efficacy and motivation, which may hinder successful implementation.^47^ Factors influencing adherence include the programme’s complexity, time constraints, perceived effectiveness, and the athlete’s understanding of the treatment rationale.^53^ Strategies to improve adherence, such as regular follow-ups, goal-setting, and technology (e.g., smartphone apps for exercise tracking), should be incorporated into SMPs to optimise outcomes.^53^

### Future directions

Given the limited evidence, uncertainty remains regarding the comparative effectiveness of different SMP combinations. More head-to-head trials are needed comparing bundled SMP interventions. Future research should also examine the feasibility and long-term adherence to SMPs for athletes with PFPS, considering the impact of training environments and lifestyle factors. Understanding factors influencing SMP adoption and maintenance is key to translating evidence into real-world sports medicine practice. This review generally provides preliminary support for multimodal SMPs for PFPS management in athletes. However, more extensive clinical trials that directly compare different combinations of self-management programme components are needed to determine the optimal treatment strategy. Implementation research is also needed to facilitate the uptake of evidence-based SMPs into the routine clinical care of athletes with PFPS, considering the various external factors that can influence treatment outcomes.

### Limitations

One of the key limitations identified in this review is the small sample sizes across several of the included studies. Small sample sizes can significantly affect the statistical power of a study, which refers to the ability to detect a true effect or difference between intervention groups. Studies with limited sample sizes are more prone to Type II errors, where real differences or effects may be present but remain undetected due to insufficient statistical power.^54^ In addition to small sample sizes, methodological differences between the included studies present another important limitation that could influence the interpretation of results. Variations in study design, intervention protocols, and outcome measures across the studies create challenges in synthesising and comparing findings. For example, differences in the specific types and intensity of exercise programs, the duration of interventions, and how outcomes such as pain reduction or functional improvement were measured can lead to inconsistent results. This heterogeneity makes it difficult to draw definitive conclusions regarding the most effective components of SMPs for athletes with PFPS.

Future research should aim to address these issues by conducting larger, more methodologically consistent trials. Increased sample sizes will enhance statistical power and improve the reliability of findings.

## Conclusion

This systematic review highlights the significant potential of self-management programmes incorporating exercise, education, and mindfulness training to reduce pain and improve functional abilities in athletes with patellofemoral pain syndrome. The evidence suggests that multimodal approaches, which address the biopsychosocial aspects of PFPS, are more effective than single-component interventions.

### Recommendations

To translate these findings into everyday practice, the following specific, actionable recommendations are tailored to different stakeholder groups.

#### Recommendations for sports therapists

1. Develop exercise regimens tailored to the specific biomechanical demands of the athlete’s sport, focusing on strengthening the hip and thigh musculature to improve patellar alignment and reduce joint loads.
2. To address muscular imbalances and abnormal patellar tracking, incorporate various exercises, including quadriceps-strengthening, hip abductor, and external rotator exercises.
3. Combine exercise therapy with trigger point therapy, manual therapy, and other physical interventions, such as orthoses and taping, to enhance outcomes.
4. Utilise mindfulness training techniques to help athletes manage pain perception and psychological factors associated with chronic pain.
5. Provide thorough education on PFPS, including the importance of load management and the rationale behind each component of the SMP.
6. Offer guidance on modifying training in response to pain levels, emphasising the importance of balancing high-impact activities with low-impact alternatives.

#### Recommendations for coaches

1. Modify training environments to reduce knee stress by incorporating more low-impact training sessions and using softer surfaces.
2. Ensure athletes access comprehensive facilities equipped with strength training equipment and physiotherapy services.
3. Collaborate closely with sports therapists to monitor the athlete’s condition and adjust training programmes to prevent symptoms from exacerbating.
4. Foster a supportive environment emphasising the importance of adhering to prescribed exercises and rehabilitation protocols.
5. Use tools such as exercise logs, mobile apps, and regular check-ins to track adherence and provide positive reinforcement for consistent effort.

#### Recommendations for coaches

1. Take an active role in self-managing PFPS by consistently performing prescribed exercises and following load management guidelines.
2. Balance high-impact sports activities with low-impact alternatives to prevent overuse injuries and promote overall joint health.
3. Maintain a nutritious diet and proper sleep hygiene to support recovery and optimise performance.

## Figures and Tables

**Table 1 t1-2078-516x-37-v37i1a18648:** The characteristics and components of the self-management programmes (SMPs) in the included studies

Author (year)	Study characteristic	

Zarei et al. (2020)	Population/sex	Female athletes
	Age range (years)	18 to 45
	Component of SMP	Exercise and dry needling
	Intervention description	The exercise programme included hamstring stretches and quadriceps strength exercises in multiple positions. - There were two groups - one received exercise only, and the other received exercise plus dry needling. - Both groups did two weekly supervised exercise sessions in the clinic with a physiotherapist for four weeks. - Both groups also did three unsupervised exercise sessions at home weekly for four weeks. - The at-home exercise sessions were unsupervised after initial instructions.
	Study design	Single-blind randomised controlled trial with follow-up
	Sample size	40, (20 per group)
	Outcome measured	Pain levels were assessed in the study using the numerical pain rating scale. The step-down and modified star excursion balance tests measured participants’ objective functional performance. Subjective functional abilities were evaluated using the validated Persian language versions of the Kujala questionnaire.
	Findings	The group versus time interaction effect was significant for all variables (p < 0.05). Both groups showed significant improvements in pain, function, and pressure pain threshold (PPT) at weeks 4 and 6 compared to baseline (p < 0.05).
	Conclusion	The research study revealed that combining trigger point therapy targeting the gluteus maximus and quadratus lumborum muscles with exercise therapy was more effective in managing patellofemoral pain compared to utilising exercise therapy alone.

Bagheri et al. (2021)	Population/sex	Female recreational runners
	Age range (years)	18 to 40
	Component of SMP	Mindfulness, training and exercise
	Intervention description	- The exercise-only group completed an 18-week exercise protocol of three sessions per week. - The mindfulness-training group received eight weeks of mindfulness training and the same 18-week exercise protocol. - The mindfulness component began four weeks before the start of the exercises. - Therefore, there was a 4-week overlap period where participants practised mindfulness concurrently with exercise during the initial portion of the programme. - After the first four weeks, the mindfulness training ceased and participants completed the remaining 14 weeks of just the exercise protocol.
	Study design	Randomised controlled clinical trial
	Sample size	30 (15 participants in each group)
	Outcome measured	A visual analogue scale (VAS) evaluated participants’ usual knee pain levels. Pain experienced during stepping and running activities was also rated with VAS. The Knee Outcome Survey was utilised to assess limitations in knee function. Psychosocial factors, including fear of movement, catastrophic thinking about pain, and coping strategies, were measured using the Tampa Scale for Kinesiophobia, Pain Catastrophising Scale, and Coping Strategies Questionnaire, respectively.
	Findings	The study found that participants in the mindfulness training group experienced significantly less pain during running and stepping activities than the exercise-only group (p-values < 0.05). The mindfulness exercise group also showed fewer functional limitations of the knee according to survey scores (p < 0.05). Additionally, the mindfulness-exercise participants reported greater perceived benefits from the treatment than those who only performed the exercises (p < 0.05). Levels of catastrophic pain were lower in the treatment group (p < 0.05).
	Conclusion	The study findings indicate that incorporating mindfulness training alongside exercise therapy can significantly augment outcomes in treating patellofemoral pain in female runners who recreationally participate in the sport.

Esculier et al. (2018)	Population/sex	Sixty-nine runners with patellofemoral pain syndrome (PFP)
	Age range (years)	18 to 45 years
	Component of SMP	Education, home exercise programme and gait retraining
	Intervention description	Each runner in the study received regular and personalised feedback on their gait. This included a 10-minute treadmill session with physiotherapist feedback on their gait at every clinic visit, ensuring their progress was closely monitored and any necessary adjustments could be made.
	Study design	A single-blind (evaluator only) parallel-group randomised control trial (RTC)
	Sample size	69 runners
	Outcome measured	The French version of the Knee Outcome Survey - Activities of Daily Living Subscale (KOS-ADLS) was utilised as the primary outcome measure to evaluate knee symptoms and functional limitations during daily activities. Knee pain levels were also measured using visual analogue scales for usual pain (VAS-U), worst pain (VAS-W) and running pain (VAS-R). Participants were given Garmin GPS-enabled running watches, and their weekly running mileages were tracked using the online Garmin Connect platform. Maximum voluntary isometric strength of the knee extensor, hip external rotator, abductor, and extensor muscles using a Medup handheld dynamometer.
	Findings	No statistically significant group versus time interactions (p < 0.447) were observed for KOS-ADLS and VASs. All three groups exhibited comparable improvements at T4, T8, and T20 compared to baseline (p < 0.05). Specifically, the exercises group demonstrated an increase in knee extension strength following rehabilitation (group × time: p < 0.001), and the gait retraining group exhibited a significant increase in step rate (+7.0%) and a decrease in average vertical loading rate (−25.4%) (group × time: p < 0.001).
	Conclusion	While gait retraining and exercises effectively enhanced their respective mechanisms, incorporating these interventions into education did not yield extra advantages in addressing symptoms and functional limitations. Comprehensive education regarding symptoms and the management of training loads should be prioritised as a fundamental element in treating runners with patellofemoral pain.

**Table 2:** Frequency, Intensity, Time and Type (FITT’s) principle of the prescribed exercises in the studies

Author (year)	Study characteristic	

Zarei et al. (2020)	Frequency	The number of repetitions increased weekly. New exercises are added each week. The study did not provide specific details on the exact number of repetitions. Progression in isometric quadriceps exercise by adding weights weekly.
	Intensity	The intensity of the exercises was not explicitly mentioned in the study.
	Time	The duration of exercise therapy was four weeks for both groups.
	Type of exercise	Isometric quadriceps exercise with progressive weight addition each week. Side-lying straight-leg raises and clamshells. Supine straight-leg raising and isometric terminal knee extension.

Bagheri et al. (2017)	Frequency	Weekly exercise frequency was increased based on individual symptoms.
	Intensity	Initial Intensity: The exercise protocol included strength-training exercises with an initial intensity of a maximum of 10 repetitions, following guidelines for strength training. Progressive Load: Participants increased the training load if they could perform exercises without aggravating knee pain or excessive fatigue, ensuring a progressive intensity approach. Individual Tailoring: A physiotherapist continuously modified the weekly exercise programme based on participants’ symptoms, indicating personalised intensity adjustments. Running Guidelines: Participants were advised to maintain patellofemoral pain (PFP) intensity at three out of 10 on the VAS while running, emphasising controlled intensity levels. Supervised Sessions: All exercises were supervised by a researcher and a physiotherapist, who ensured proper form and intensity management during training sessions.
	Time	Three 60- to 90-minute sessions per week over 18 weeks.
	Type of exercise	The exercise protocol included 13 exercises: 6 stretching, 7 strength/balance. The protocol included warm-up, cool-down, jogging, dynamic exercises, and rest intervals. The initial intensity for strength-training exercises was a maximum of 10 repetitions.

Esculier et al. (2018)	Frequency	The exercise programme was designed to be performed weekly, indicating a frequency of at least once a week.
	Intensity	Initial Intensity: The exercise protocol included strength-training exercises with an initial intensity of 10 repetitions. Progressive Load: Participants increased the training load if they could perform exercises without aggravating knee pain or excessive fatigue. A physiotherapist continuously modified the weekly exercise programme based on participants’ symptoms, indicating personalised intensity adjustments. Running Guidelines: Participants were advised to maintain a specific intensity level on the Visual Analog Scale (VAS) while running, emphasising controlled exercise intensity.
	Time	Participants attended five physiotherapy sessions over an 8-week period, with sessions scheduled at weeks 1, 2, 3, 5, and 7.
	Type of exercise	Participants engaged in a structured exercise programme that included strength-training exercises targeting the gluteus medius (GM) and quadratus lumborum (QL) muscles.

**Table 3:** The methodological quality of the included study as rated with the Physiotherapy Evidence Database (PEDro) scale

Authors	Eligibility criteria	Random allocation	Concealed allocation	Baseline comparability	Patient blinding	Therapist blinding	Assessor blinding	Less than 15% dropout	Between-group statistical comparisons	Intention-to-treat analysis	Point measures and variability data	Total

Zarei et al. (2020)	1	1	1	1	1	1	1	0	0	1	1	10
Esculier et al. (2017)	1	1	1	1	0	0	1	0	0	1	1	7
Bagheri et al. (2021)	1	1	1	1	0	0	1	0	0	1	1	7

## Data Availability

Data sharing will be available on request.
